# Evaluation of adjuvant chemoradiation therapy for ampullary adenocarcinoma: the Johns Hopkins Hospital - Mayo Clinic collaborative study

**DOI:** 10.1186/1748-717X-6-126

**Published:** 2011-09-28

**Authors:** Amol K Narang, Robert C Miller, Charles C Hsu, Sumita Bhatia, Timothy M Pawlik, Dan Laheru, Ralph H Hruban, Jessica Zhou, Jordan M Winter, Michael G Haddock, John H Donohue, Richard D Schulick, Christopher L Wolfgang, John L Cameron, Joseph M Herman

**Affiliations:** 1 Department of Radiation Oncology, Johns Hopkins University School of Medicine, Baltimore, MD, USA; 2 Department of Radiation Oncology, The Mayo Clinic, Rochester, MN, USA; 3 Department of Radiation Oncology, University of California, San Francisco, San Francisco, CA, USA; 4 Department of Surgery, Johns Hopkins University School of Medicine, Baltimore, MD, USA; 5 The Sol Goldman Pancreatic Research Center, Johns Hopkins University School of Medicine, Baltimore, MD, USA; 6 Department of Oncology, Johns Hopkins University School of Medicine, Baltimore, MD, USA; 7 Department of Pathology, Johns Hopkins University School of Medicine, Baltimore, MD, USA; 8 Department of Surgery, The Mayo Clinic, Rochester, MN, USA

**Keywords:** ampullary, carcinoma, adjuvant, chemoradiation, resectable

## Abstract

**Background:**

The role of adjuvant chemoradiation therapy for ampullary carcinoma is unknown. Previous literature suggests that certain populations with high risk factors for recurrence may benefit from adjuvant chemoradiation. We combined the experience of two institutions to better delineate which patients may benefit from adjuvant chemoradiation.

**Methods:**

Patients who underwent curative surgery for ampullary carcinoma at the Johns Hopkins Hospital (n = 290; 1992-2007) and at the Mayo Clinic (n = 130; 1977-2005) were reviewed. Patients with <60 days of follow-up, metastatic disease at surgery, or insufficient pathologic data were excluded. The final combined study consisted of 186 patients (n = 104 Johns Hopkins, n = 82 Mayo). Most patients received 5-FU based chemoradiation with conformal radiation. Cox proportional hazards models were used for survival analysis.

**Results:**

Median overall-survival was 39.9 months with 2- and 5-year survival rates of 62.4% and 39.1%. On univariate analysis, adverse prognostic factors for overall survival included T3/T4 stage disease (RR = 1.86, p = 0.002), node positive status (RR = 3.18, p < 0.001), and poor histological grade (RR = 1.69, p = 0.011). Patients who received adjuvant chemoradiation (n = 66) vs. surgery alone (n = 120) showed a higher rate of T3/T4 stage disease (57.6% vs. 30.8%, P < 0.001), lymph node involvement (72.7% vs. 30.0%, P < 0.001), and close or positive margins (4.6% vs. 0.0%, P = 0.019). Five year survival rates among node negative and node positive patients were 58.7% and 18.4% respectively. When compared with surgery alone, use of adjuvant chemoradiation improved survival among node positive patients (mOS 32.1 vs. 15.7 mos, 5 yr OS: 27.5% vs. 5.9%; RR = 0.47, P = 0.004). After adjusting for adverse prognostic factors on multivariate analysis, patients treated with adjuvant chemoradiation demonstrated a significant survival benefit (RR = 0.40, P < 0.001). Disease relapse occurred in 37.1% of all patients, most commonly metastatic disease in the liver or peritoneum.

**Conclusions:**

Node-positive patients with resected ampullary adenocarcinoma may benefit from 5-FU based adjuvant chemoradiation. Since a significant proportion of patients develop metastatic disease, there is a need for more effective systemic treatment.

## Background

Although carcinoma of the ampulla of Vater is a rare malignancy with an overall incidence of 6 in 1 million, it is the second most common periampullary cancer, comprising 6-20% of malignancies in this region [1-3]. Compared to pancreatic adenocarcinoma, ampullary cancer is associated with a higher likelihood of resectability and a more favorable prognosis. Whereas patients with resectable pancreatic adenocarcinoma show a 5-year survival of only 20%, most retrospective reviews of ampullary cancer over the past two decades have reported 5-year survival between 30-40% [4-11]. The earlier appearance of obstructive symptoms, more favorable histology, and a decreased inclination for lymphatic or perineural invasion have all been cited as potential explanations for the better outcomes with ampullary carcinoma [12].

Pancreaticoduodenectomy (PD) remains the only possible curative treatment for patients with pancreatic or ampullary cancer, but the role of adjuvant therapy remains controversial. In the United States, postoperative adjuvant chemoradiation (CRT) has been used for pancreatic cancer based on evidence suggesting improved survival [4,13,14]. Whether these results can be extrapolated to resected ampullary carcinoma has been an area of active debate. A 1999 randomized controlled trial by the European Organization for Research and Treatment of Cancer (EORTC) examined post-operative 5-fluorouracil (5-FU) based CRT in patients with pancreatic head or other periampullary malignancies. This study demonstrated no survival benefit in patients with periampullary cancer at 2 or 5 years, but the number of patients with ampullary carcinoma was small, most of whom had favorable prognostic factors [14]. More recently, a retrospective review from the MD Anderson Cancer Center showed a borderline significant improvement in survival with CRT in a subset of patients with advanced tumor stage (T3/T4), while a study from the Mayo Clinic found a survival benefit in patients with pathologic lymph node involvement [15,16]. A third review from the Johns Hopkins Hospital (JHH) also suggested a potential survival benefit from CRT in patients with resected ampullary carcinoma who had lymph node involvement, although this finding was not statistically significant (p = 0.092) [17]. While these studies indicate that certain subsets of patients with ampullary carcinoma may benefit from postoperative CRT, they are limited by the small number of patients analyzed. In the present study, we combine the experience of two of the aforementioned institutions, namely the Johns Hopkins Hospital and the Mayo Clinic, to compare surgery followed by modern conformal 5-FU based adjuvant CRT with surgery alone for patients with resectable carcinoma of the ampulla of Vater.

## Methods

### Study design and participants

The study was approved by the institutional review boards of the Mayo Clinic, Rochester, MN, and the Johns Hopkins Hospital, Baltimore, MD. The study cohort was drawn from all patients who underwent curative surgery for ampullary carcinoma at the Johns Hopkins Hospital between 1992 and 2007 (n = 290, prospectively collected) and the Mayo Clinic from 1977 to 2005 (n = 130, retrospectively collected). Cancer of the ampulla of Vater was defined as adenocarcinoma directly centered on or associated with an *in situ *carcinoma of the ampulla, papilla, or both, as evidenced by review of the final pathology report. Patients with cancers arising from the duodenum, pancreatic head, or common bile duct were not eligible. Patients who were referred to outside institutions for adjuvant therapy or follow-up care were excluded because information regarding the details of their outcomes or whether they received adjuvant treatment was unavailable (n = 156). Individuals who died within 60 days of surgery (n = 6), had less than 60 days of follow-up (n = 6), or had evidence of metastatic disease at surgery (n = 11) were excluded as well. Those missing information on T-stage, tumor size, margin status, node status, or histologic grade (n = 55) were also not analyzed. The final study population contained 186 patients (n = 104 JHH, n = 82 Mayo).

All patients received preoperative staging by one or more of the following modalities: abdominal and pelvic-computed tomography (CT), endoscopic retrograde cholangiopancreatography (ERCP), endoscopic ultrasonography (EUS), and percutaneous transhepatic cholangiography or percutaneous biliary drainage (PTC/PBD). The majority of patients (77%) received both CT and ERCP. Laboratory tests included a full blood count, serum electrolytes, creatinine, urea, liver transaminases, alkaline phosphatase, total bilirubin, carcinoembryonic antigen (CEA), and carbohydrate antigen 19-9 (CA19-9).

Patient demographic and pathologic data are outlined in Table 1. Of the 186 patients in the study, 109 (59%) were male. Median age was 68 years (range 29-90 years). Pathologic data is limited to those variables potentially conferring poor prognosis, including T-stage, tumor size, lymph node status, histologic grade, and margin status.

**Table 1:** Baseline Characteristics between Treatment Groups

	Observation Only N = 120	Adjuvant Chemoradiation (CRT) Therapy N = 66	P-Value
**DEMOGRAPHIC**			

**Age at Surgery (yr)**			
Mean, (SD*)	68.9 (11.6)	62.0 (10.8)	<0.001
Median (Range)	71.3 (28.7-90.3)	63.3 (29.3-81.5)	

**Gender**			
Male, No. (%)	66 (55.0)	43 (65.2)	0.179

**Institution**			
Mayo Clinic	63 (52.5)	19 (28.8)	0.002
Johns Hopkins Hospital (JHH)	57 (47.5)	47 (71.2)	

**TUMOR CHARACTERISTCS**			

**T Stage**			
1	37 (30.8)	8 (12.1)	0.002
2	46 (38.3)	20 (30.3)	
3	33 (27.5)	34 (51.5)	
4	4 (3.3)	4 (6.1)	

**Tumor Diameter**			
< 3 cm	81 (67.5)	39 (59.1)	0.251
≥ 3 cm	39 (32.5)	27 (40.9)	

**Nodal Status**			
N0	84 (70.0)	18 (27.3)	<0.001
N1	36 (30.0)	48 (72.3)	

**Histologic Grading**			
1	8 (6.7)	0 (0.0)	0.053
2	52 (43.3)	25 (37.9)	
3	60 (50.0)	41 (62.1)	

**Surgical Margins**			
Positive	0 (0.0)	3 (4.6)	0.019
Negative	120 (100.0)	63 (95.4)	

*SD = standard deviation

### Surgery

Patients underwent either a pylorus-preserving or classic pancreaticoduodenectomy (PD). A pylorus-preserving PD included resection of the head and uncinate process of the pancreas, distal bile duct, all but the most proximal duodenum, and gallbladder, when present. In a classic PD, the antrum of the stomach was also resected. At JHH, 82.5% of patients underwent a pylorus-preserving PD, while these data were unavailable for patients treated at the Mayo Clinic. All pathology specimens were reviewed by either a pathologist at JHH or centrally at the Mayo Clinic, and patients were restaged according to American Joint Committee on Cancer (AJCC) guidelines, sixth edition. Pathologic data regarding T stage, tumor size, histologic grade, lymph node involvement, lymphovascular invasion, perineural invasion, and surgical margins were recorded. Lymph nodes were considered positive if the resection specimen contained metastatic carcinoma in any of the lymph nodes, whether they were involved by direct extension or contiguous with the primary tumor. At the Mayo Clinic, margin status was determined by the presence of carcinoma at the final pancreatic neck, uncinate process, bile duct, duodenal, or retroperitoneal soft tissue margin. At JHH, resection margins were considered positive if the carcinoma was close to (within 1 mm) or present at these margins.

### Chemoradiation regimen

Of the 186 patients in this study, 120 (64.5%) received surgery alone, while 66 (35.5%) were given adjuvant CRT. In patients receiving adjuvant therapy, radiation treatments were administered with a 3-field coplanar approach (7.6%), 4-field coplanar approach (78.8%), 5-field non-coplanar approach (3.0%), or intensity modulated radiation therapy (IMRT, 10.6%). A total of 45 Gy was generally delivered to the ampullary tumor bed (based on preoperative images), surgical anastomoses (hepatojejunostomy, pancreaticojejunostomy) and adjacent regional lymph nodes (proximal celiac and superior mesenteric). Additional radiation (5-15 Gy) was administered to the tumor bed/area of involved margins and anastomoses paying careful attention to the dose to the small bowel. The median total dose was 50.4 Gy (range 37.8-50.4 Gy). Radiation was given in consecutive, daily fractions except for 7 patients (10.6%) who underwent a two week planned break in therapy as part of a treatment protocol investigating split-course chemoradiation. In this protocol, patients received two weeks of 5-fluorouracil based chemoradiation, a two week treatment break, and two additional weeks of 5-fluorouracil based chemoradiation, followed by 5-fluorouracil based maintenance chemotherapy. No patient received neoadjuvant or intraoperative radiation. Concurrent chemotherapy most commonly consisted of 5-fluorouracil (95.5%), although three patients (4.5%) received gemcitabine. Maintenance chemotherapy was given to 37.9% of patients in the form of single-agent 5-fluorouracil (15.2%), single-agent gemcitabine (19.7%), or combination gemcitabine with either cisplatin/erlotinib (1.5%) or capecitabine (1.5%). All patients who received maintenance chemotherapy were treated at JHH. Note that patients were not excluded from our analysis based on the concurrent or maintenance chemotherapeutic agents that were administered given the lack of clear evidence supporting a specific regimen. None of the patients in this study were treated with adjuvant chemotherapy alone.

### Statistical Analysis

Statistical analysis was performed using STATA, version 9 (Stata, College Station, TX). Summary statistics for continuous and dichotomous variables are provided. In constructing dichotomous variables, thresholds were defined in accordance with the literature [15-18]. The distribution of prognostic variables between treatment groups was compared using Pearson's chi-squared test. The primary outcome variable was overall survival (OS), defined as the time from surgical resection to death. Survival time was censored at date of last follow up if death had not occurred. Univariate analysis was conducted using the log-rank test to examine risk factors and associations with mortality. Median OS was estimated within each risk group and by adjuvant treatment. The proportion of individuals surviving up to 2 and 5 years was calculated using life tables and stratified by treatment group to assess for a significant difference using the log-rank test. Proportional hazards models were used to examine the association of adjuvant treatment, baseline patient characteristics, and pathologic data with mortality. To explore the independent association of adjuvant therapy and OS, multivariate analysis was performed, adjusting for possible confounders, namely age, sex, institution, tumor stage, tumor size, lymph node status, and histologic differentiation. Margin status was not included in multivariate analysis due to a paucity of patients with close or positive margins (n = 3). Survival curves were estimated with Kaplan-Meier techniques.

## Results

At the time of analysis, 82 patients (44.1%) were still alive while 104 patients (55.9%) had died. Progression of disease was the cause of death for 58 patients (55.2%), while the remaining 46 deaths (44.8%) were from unknown or other causes. Median follow-up time for surviving patients was 31.7 months (range 2.0 - 160.1 months).

As displayed in Table 1, when compared with patients who were treated with surgery alone, those patients who received adjuvant CRT were significantly younger (62.0 vs. 68.9, p < 0.001), were more likely from JHH (71.2% vs. 47.5%, p = 0.002), had more advanced T-stage (T3/T4: 57.6% vs. 30.8%, p = 0.002), and showed more frequent pathologic lymph node involvement (72.3% vs. 30.0%, p < 0.001). Patients in the CRT group also more frequently had close or positive surgical margins (4.6% vs. 0.0%, p = 0.019), although only three patients in the entire sample had close or positive margins, all of whom were given CRT. Histologic grade, while not significantly different between treatment groups, did show a trend towards poorer differentiation amongst patients given CRT (grade 3: 62.1% vs. 50.0%, p = 0.053). Neither tumor size nor gender was associated with the type of treatment that the patient received. Additionally, when patient demographics and tumor characteristics were stratified by institution, there was no significant difference between the JHH and Mayo cohorts for any of these factors (results not shown).

**Figure 1:**
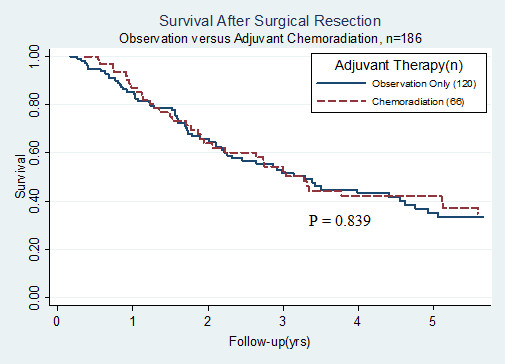
**Survival following pancreaticoduodenectomy stratified by type of adjuvant therapy**. Kaplan-Meier curves comparing overall survival between patients who received adjuvant chemoradiation (n = 66) and those treated with surgery alone (n = 120). Adjuvant therapy was not significantly associated with improved overall survival (p = 0.839) on univariate analysis.

**Figure 2:**
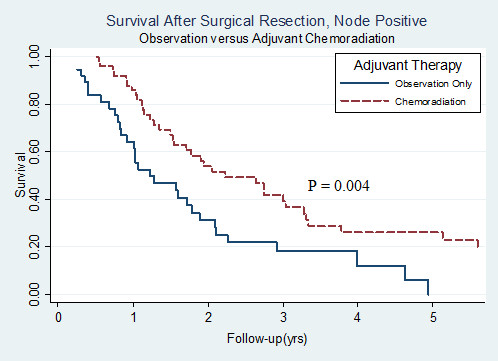
**Survival following pancreaticoduodenectomy in node positive patients stratified by type of adjuvant therapy**. Kaplan-Meier curves comparing overall survival amongst node-positive patients between patients who received adjuvant chemoradiation (n = 48) and those treated with surgery alone (n = 36). In node-positive patients, adjuvant therapy was significantly associated with improved overall survival (p = 0.004) on univariate analysis.

Median overall survival (mOS) for all patients was 39.9 months (95% CI: 29.5 - 54.7 months) with a 2-year and 5-year survival of 62.4% and 39.1%, respectively. As displayed in Table 2, on univariate analysis, lymph node involvement had the strongest association with decreased overall survival (mOS: 23.0 vs. 79.4 months, RR 2.11 - 4.78, p < 0.001). Advanced T-stage and poor histologic differentiation were also significantly associated with poor prognosis. Specifically, tumors classified as T3/T4 showed significantly worse overall survival compared with T1/T2 disease (mOS: 27.0 vs. 55.4 months, RR 1.26 - 2.75, p = 0.002), as did grade 3 histology when compared with grade 1 or 2 disease (mOS: 32.1 vs. 60.0 months, RR 1.13 - 2.53, p = 0.011). Age, gender, institution, tumor size, and margin status were not predictive of overall survival. Furthermore, as illustrated in Figure 1, adjuvant treatment with CRT was not significantly associated with overall survival when compared with surgery alone (median survival 39.9 vs. 40.1 months, RR 0.64 - 1.43, p = 0.839) using univariate analysis. As shown in Table 3, when patients were stratified into eight risk groups, and survival by treatment type was compared within each subgroup, the only patients who showed a significant difference in median survival between adjuvant CRT and surgery alone were those with pathologic lymph node involvement (mOS: 32.1 vs. 15.7 months, p = 0.004). In node-positive patients, adjuvant CRT resulted in a 5-year survival of 27.5%, while surgery alone led to a 5-year rate of only 5.9%. Figure 2 compares the survival curves by treatment type for node-positive patients. Median survival was also higher in node negative patients receiving adjuvant CRT (mOS: 103.2 vs. 61.6 months), but the difference was not statistically significant (p = 0.122).

**Table 2:** Associations of Overall Survival with Patient Tumor and Treatment Characteristics

	No. (%)	2-Year Survival, %	5-year Survival, %	Median Survival, months	Univariate RR (95% CI)	P-value
**Age, yrs**						

< 75	137 (73.7)	66.5	41.2	40.6	1.00	0.281

≥ 75	49 (26.3)	66.7	32.5	35.5	1.27 (0.82 - 1.98)	

**Gender**						

Female	77 (41.4)	67.4	37.4	39.9	1.00	0.954

Male	109 (58.6)	66.0	40.4	36.5	0.99 (0.67 - 1.47)	

**Institution**						

Mayo Clinic	82 (44.1)	68.6	39.9	40.6	1.00	0.350

JHH	104 (55.9)	64.8	38.9	36.9	1.20 (0.81 - 1.78)	

**T stage**						

1/2	111 (59.7)	75.0	46.3	55.4	1.00	0.002

3/4	75 (40.3)	54.1	28.1	27.0	1.86 (1.26 - 2.75)	

**Tumor Size**						

≤ 3 cm	120 (64.5)	72.5	39.3	40.1	1.00	0.838

> 3 cm	66 (35.5)	56.5	38.3	35.5	1.04 (0.70 - 1.55)	

**Node Status**						

Negative	102 (54.8)	84.1	58.7	79.4	1.00	<0.001

Positive	84 (45.2)	47.2	18.4	23.0	3.18 (2.11 - 4.78)	

**Histology**						

Grade 1/2	85 (45.7)	75.8	49.9	60.0	1.00	0.011

Grade 3	101 (54.3)	59.1	30.6	32.1	1.69 (1.13 - 2.53)	

**Margin Status**						

Negative	183 (98.4)	66.6	39.3	39.9	1.00	0.493

Positive	3 (1.6)	33.3	0.0	33.3	1.49 (0.47 - 4.72)	

**Adjuvant Treatment**						

None	120 (64.5)	67.3	37.2	40.1	1.00	0.839

CRT*	66 (35.5)	65.3	42.1	39.9	0.96 (0.64 - 1.43)	

*CRT = chemoradiation

**Table 3:** Survival between Treatment Groups by Patient, Tumor, and Treatment Characteristics

	No. of Patients, (%)		Overall Survival, Median, mo			5-year Survival, Percent	
	**Observation**	**Adjuvant CRT**	**Observation**	**Adjuvant CRT**	**P-value**	**Observation**	**Adjuvant CRT**

**ALL PATIENTS**	120 (64.5)	66 (35.5)	39.9	40.1	0.839	37.2	42.1

**Age, yrs**							

< 75	79 (57.7)	58 (42.3)	41.3	40.6	0.913	37.9	45.9

≥ 75	41 (83.7)	8 (16.3)	35.5	33.3	0.939	36.0	25.0

**Gender**							

Female	54 (70.1)	23 (20.9)	42.7	32.1	0.162	40.8	29.3

Male	66 (60.6)	43 (39.4)	32.2	46.0	0.238	33.9	48.4

**Institution**							

Mayo	63 (76.8)	19 (23.2)	38.2	62.4	0.599	35.5	51.3

Hopkins	57 (54.8)	47 (45.2)	41.7	36.5	0.890	40.0	37.0

**Histology**							

Grade 1/2	60 (70.6)	25 (29.4)	53.6	62.2	0.328	47.1	56.6

Grade 3	60 (59.4)	41 (40.6)	34.9	27.1	0.985	27.5	34.1

**Node**							

Negative	84 (82.4)	18 (17.7)	61.6	103.2	0.122	52.4	87.1

Positive	36 (42.9)	48 (57.1)	15.7	32.1	0.004	5.9	27.5

**Tumor Stage**							

T1/T2	83 (74.8)	28 (25.2)	41.3	87.5	0.172	41.7	56.8

T3/T4	37 (49.3)	38 (50.7)	27.0	25.0	0.873	27.5	28.7

**Tumor Size**							

≤ 3 cm	81 (67.5)	39 (32.5)	41.3	36.5	0.797	38.8	40.0

> 3 cm	39 (59.1)	27 (40.9)	27.0	40.6	0.496	33.9	44.0

**Margin status**							

Negative	120 (65.6)	63 (34.4)	40.1	39.9	0.754	37.2	42.7

Positive	0 (0.0)	3 (100.0)	N/A	33.3	N/A	N/A	33.3

*CRT = chemoradiation

As displayed in Table 4, on multivariate analysis, adjuvant CRT was significantly associated with improved overall survival (RR 0.25 - 0.67, p < 0.001), when adjusted for age, gender, institution, T-stage, tumor size, node status, and grade. Additionally, lymph node involvement was the only other variable associated with overall survival on multivariate analysis, with node positive patients experiencing significantly increased risk of death (RR 2.50 - 7.17, p < 0.001).

**Table 4:** Multivariate Cox Proportional Hazards Survival Analysis of Adjuvant Chemoradiation therapy and Overall Survival

	RR (95% CI)	P-value
**Age, yrs**		

< 75	1.00	0.755

≥ 75	0.93 (0.57 - 1.50)	

**Gender**		

Female	1.00	0.402

Male	0.84 (0.56-1.26)	

**Institution**		

Mayo	1.00	0.222

Hopkins	1.30 (0.85-1.99)	

**Tumor Stage**		

T1/T2	1.00	0.317

T3/T4	1.24 (0.81-1.91)	

**Tumor Size**		

**≤ **3 cm	1.00	0.391

> 3 cm	1.20 (0.79-1.80)	

**Node**		

Negative	1.00	<0.001

Positive	4.29 (2.5-7.17)	

**Histology**		

Grade 1/2	1.00	0.191

Grade 3	1.35 (0.86-2.41)	

**Adjuvant Treatment**		

Observation	1.00	<0.001

Adjuvant CRT*	0.41 (0.25 - 0.67)	

*CRT = chemoradiation

Of the 66 patients who underwent adjuvant CRT, 41 (62.1%) experienced some form of toxicity during therapy. The most common toxicities reported were nausea (25.8%), diarrhea (16.7%), weight loss (9.1%), fatigue (9.1%), and epigastric pain (7.6%). While side effects tended to be mild in nature, treatment-related toxicity did lead to an interruption of therapy in 8 patients (12.1%). Grade 3 toxicities were reported in two patients from the Mayo Clinic who suffered from myelosuppression and sepsis respectively. The grade of toxicity for patients treated at JHH was unavailable, although no patient from JHH was hospitalized for radiation-associated toxicity. There were no known treatment related deaths.

Sixty-nine patients (37.1%) experienced a recurrence by the end of follow-up. The most common pattern of initial recurrence was distant metastasis without local relapse, which was seen in 49 patients (26.3%). Thirteen patients (7.0%) had both local and metastatic disease at initial relapse. Only 7 patients (3.8%) presented with local recurrence without evidence of metastatic spread, of which 6 had not been given adjuvant therapy. The distribution in patterns of initial recurrence between treatment groups is summarized in Table 5. Overall, the liver was the most common site of metastasis, with 24.7% of all patients and 36.5% of those patients who died harboring disease in the liver. The peritoneum was the second most common site of metastasis, present in 5.9% of all patients and 9.5% among those patients who died. Lung metastases were found in 4.8% of all patients and 6.7% of patients who died, making it the third most common site of distant spread.

**Table 5:** Initial Sites of First Recurrence by Treatment Group

	No Recurrence	Local Recurrence	Distant Recurrence	Local & Distant	Overall Recurrences	Total Patients
**CRT**	31 (47.0%)	1 (1.5%)	23 (34.8%)	11 (16.7%)	35 (53.0%)	66

**No CRT**	86 (71.7%)	6 (5.0%)	26 (21.7%)	2 (1.7%)	34 (28.3%)	120

**Total**	118 (63.4%)	7 (3.8%)	49 (26.3%)	12 (6.5%)	68 (36.6%)	186

## Discussion

This combined series of patients with ampullary carcinoma represents the largest study to date that demonstrates an overall survival benefit in patients receiving adjuvant chemoradiation following surgical resection when controlling for adverse prognostic factors. After adjusting for institution, patient demographics such as age and gender, and disease characteristics such as tumor stage, tumor size, nodal involvement, and histology, patients treated with adjuvant CRT experienced enhanced survival (HR = 0.41, 95% CI: 0.25-0.67, p < 0.001). This series also confirms improved outcomes in patients with ampullary carcinoma when compared with pancreatic cancer, with a median survival of 39.9 months and two and five-year survival rates of 62.4% and 39.1% respectively.

Pancreaticoduodenectomy is the preferred surgical approach for carcinoma of the ampulla of Vater that is amenable to resection [19]. However, similar to pancreatic cancer, the role of post-operative adjuvant therapy remains undefined. While prognosis for resectable ampullary carcinoma is considerably better than for pancreatic cancer, patients with node positive disease have poor survival and appear to benefit from adjuvant therapy [20,21]. A number of reports, mostly consisting of single institution series, have established adverse prognostic factors, including extent of local invasion, status of surgical margins, presence of nodal metastasis, and histologic grade, all of which predict for overall survival as well as local and distant disease [5-9,22-29]. In these cohorts, nodal involvement has been a particularly strong predictor of poor outcomes, with 5-year survival rates following PD ranging from 64-80% in patients with node-negative disease and 17-50% in patients with node-positive disease. A more recent population-based analysis of roughly 4,000 patients with ampullary carcinoma was conducted using the National Cancer Institute's Surveillance, Epidemiology, and End Results database [30]. Outcomes were slightly worse than the aforementioned series from specialized cancer centers but were highly dependent on nodal metastasis (5 year survival: 47.6% vs. 21.0%).

High rates of relapse along with identification of adverse prognostic factors have led to exploration of adjuvant chemoradiation for "high risk" ampullary carcinoma, although the literature in this area remains sparse. Willett et al. first reported a trend towards improved local control with no improvement in overall survival when adjuvant 5-FU based chemoradiation was given to a small cohort of patients with high risk features, defined as invasion of the pancreas, nodal metastasis, positive margins, or poor histology [31]. A subsequent review by Mehta et al. reported a favorable 3-year actuarial survival rate of 44% using adjuvant 5-FU based chemoradiation in patients with large tumor size, nodal involvement, positive surgical margins, poor histology, or neurovascular invasion [32]. Similarly, Lee et al. achieved superior disease-free survival in patients with advanced tumor stage (T3/T4) or positive nodes receiving adjuvant chemoradiation [33]. On multivariate analysis, adjuvant therapy was also a significantly favorable factor for the entire cohort (HR: 0.16, p = 0.030). However, less than twenty patients received adjuvant therapy in each of these studies, making it difficult to derive convincing conclusions.

More recently, three retrospective studies from institutions that treat high volumes of periampullary malignancies reviewed their experience with adjuvant CRT for ampullary carcinoma. Krishnan et al. examined 96 patients, 54 of whom had received adjuvant CRT consisting of either preoperative radiation to a median dose of 45 Gy or postoperative radiation to a median dose of 50.4 Gy, with concurrent 5-FU or capecitabine [15]. Patients with advanced T-stage (T3/T4) who were treated with CRT showed a borderline significant increase in survival (mOS: 35.2 vs. 16.5 months, p = 0.06). Similarly, a JHH review of 111 patients identified a trend towards improved survival with adjuvant CRT among those patients with nodal metastasis (mOS: 30.0 vs. 21.6 months, p = 0.092) [17]. Postoperative therapy in this study consisted of a median radiation dose of 50.4 to the tumor bed and regional nodes with concurrent 5-FU or capecitabine. Furthermore, a statistically significant difference in survival among patients with lymph node involvement treated with adjuvant CRT was found in a previous study from the Mayo Clinic (mOS: 3.4 vs. 1.6 years, p = 0.02) [16]. Note, however, that in none of these studies was adjuvant CRT associated with increased survival on multivariate analysis.

In the present series, adjuvant therapy was not found on univariate analysis to be associated with increased survival when compared to surgery (mOS: 39.9 vs. 40.1 months, p-0.839), as summarized in Table 2 and illustrated in Figure 1. This lack of survival benefit is likely a result of the imbalance in adverse prognostic factors between treatment groups. In this series, nodal metastasis (p < 0.001), advanced T stage (p = 0.002), and poorly differentiated histology (p = 0.011) were all significantly associated with decreased survival. While margin status was not a predictor of survival (p = 0.493), margin status is widely considered to be a poor prognostic factor, and its lack of association with survival in this study may be attributable to the small number of patients with close or positive margins (n = 3). Regardless, the cohort that received CRT had a significantly higher proportion of patients with advanced T-stage (p = 0.002), pathologic lymph node involvement (p < 0.001), and positive surgical margins (p = 0.019), and a borderline statistically significant trend towards poorer histologic grade (p = 0.053). The fact that survival was comparable between treatment groups despite these discrepancies suggests the potential benefit of adjuvant CRT, particularly amongst high risk populations.

Moreover, when baseline demographic and treatment-related characteristics were adjusted for on multivariate analysis, a significant association between adjuvant therapy and improved survival appeared. Indeed, this study represents the second reported survival benefit from adjuvant CRT found on multivariate analysis, albeit with a much larger cohort than was analyzed in the aforementioned study by Lee et al [33]. Interestingly, when patients were stratified by baseline demographic and disease-related characteristics, no subgroup showed a significant survival benefit from adjuvant CRT except for patients with nodal metastasis, who experienced a large difference in median survival (mOS: 32.1 vs. 17.5 months, p = 0.004). As suggested in multiple previous studies, node-positive patients were found to carry a very poor prognosis on both univariate analysis (p < 0.001) and multivariate analysis (p < 0.001), with a median survival of only 18.4 months. The fact that node-positive patients who were not treated with adjuvant CRT showed a dismal 5-year survival rate of only 5.9% indicates that this group may be particularly suited for post-operative therapy. Moreover, while the effect of adjuvant therapy in node negative patients did not reach statistical significance, the absolute difference in survival (mOS: 103.2 vs. 61.6 months) is noteworthy and reminiscent of the CONKO-001 trial in which node negative pancreatic cancer patients experienced superior survival with adjuvant chemotherapy [34].

While good local control was achieved in this study, nearly a third of patients suffered from distant relapse, and roughly 90% of recurrences were attributable in part to metastatic disease. Consistent with the literature, the most common sites of metastasis were the liver and peritoneum. Overall, progression of disease led to more than half of the deaths in the cohort, with nearly one third of patients who died harboring disease in the liver. The prevalence of metastatic disease suggests the need for more effective systemic therapy, particularly in high risk patients. Unfortunately, there is even less information regarding appropriate type and duration of chemotherapeutic agents when incorporated with radiation for ampullary cancer. Furthermore, the role of adjuvant chemotherapy alone is an area that has been largely understudied, a remnant of borrowed U.S. practice patterns supporting adjuvant CRT for resected pancreatic cancer. A Japanese study of adjuvant mitomycin C and 5-FU for pancreaticobiliary carcinomas found no overall or disease-free survival benefit in a subset of 24 patients with ampullary carcinoma when compared to surgery alone [35]. More recent and robust results from the European Study Group for Pancreatic Cancer (ESPAC) - 3(v2) trial also showed no difference in survival in 304 patients with resected ampullary cancer who were randomized to 5-FU/folinic acid, gemcitabine, or observation [36]. Combination chemotherapy may provide better results, as a randomized control trial comparing gemcitabine and cisplatin versus gemcitabine alone in 410 patients with locally advanced or metastatic biliary or ampullary cancers did show superior survival with the combination regimen (mOS: 11.7 vs. 8.1 months, p < 0.001), although it should be acknowledged that only 5% of tumors in the study had an ampullary origin [37]. Of note, no study has directly compared adjuvant chemotherapy alone with adjuvant chemoradiation.

Given the retrospective nature of this study and wide time period over which this study spanned, our findings are limited by the variability in treatment regimens and the potentially unequal distribution of confounding factors in patient selection between treatment groups. Certainly, patients in our study were subject to different operative methods, radiation plans, and chemotherapeutic agents, which we were unable to control for due to incomplete information or insufficient power. Additionally, this study has shown that patients selected for adjuvant CRT at the Johns Hopkins Hospital and the Mayo Clinic possessed more adverse prognostic factors than those treated with surgery alone. While several high risk characteristics were adjusted for in our analysis, other variables that were not taken into account include performance status and weight loss, both of which may be correlated with disease outcomes. Since patients who received adjuvant CRT were significantly younger, it is easy to imagine that healthier patients were more likely offered adjuvant treatment. The retrospective nature of our study may have also compromised our ability to accurately capture certain information such as the toxicity data, which was lower when compared to prior experience [14]. Furthermore, variations in institutional protocols regarding treatment delivery can be a source of bias in studies analyzing data from multiple sites, but it should be noted that while the distribution of treatment type did in fact vary by institution, there was no association between institution and survival on univariate or multivariate analysis, and institution did not affect outcomes when stratified by treatment type. Another limitation was the number of patients excluded for either missing data (i.e. stage or nodal status) or because they were lost to follow-up. It is probable that follow-up was not consistent among treatment groups, with patients receiving adjuvant therapy likely showing better follow-up. The number of patients lost to follow along with the number of patients alive at time of analysis resulted in a low number of documented recurrences. Ideally, we would have been able to examine the association between adjuvant therapy and patterns of recurrence, but the low number of recurrences prevented the possibility of meaningful analysis. Nevertheless, this study combines the experience of two high volume institutions to allow for the largest series to date that has examined the role of adjuvant therapy following surgery for ampullary cancer.

## Conclusions

Lymph node involvement, advanced tumor stage, and poor histology are adverse prognostic factors associated with poor survival in patients with ampullary carcinoma. The addition of adjuvant chemoradiation likely improves survival in patients with high risk disease, particularly in those with lymph node involvement. Whether all patients with resectable ampullary carcinoma should be treated with adjuvant chemoradiation is subject to debate. Certainly, better systemic therapy is necessary to improve the high rate of distant metastasis found in this population.

## List of abbreviations

PD: pancreaticoduodenectomy; CRT: chemoradiation therapy; EORTC: European Organization for Research and Treatment of Cancer; 5-FU: 5-fluorouracil; JHH: Johns Hopkins Hospital; CT: computed tomography; ERCP: endoscopic retrograde cholangiopancreatography; EUS: endoscopic ultrasonography; PTC: percutaneous transhepatic cholangiography; PBD: percutaneous biliary drainage; CEA: carcinoembryonic antigen; CA19-9: carbohydrate antigen 19-9; AJCC: American Joint Committee on Cancer; IMRT: intensity modulated radiation therapy.

## Competing interests

The authors declare that they have no competing interests.

## Authors' contributions

AKN participated in the analysis and interpretation of data and drafting of the manuscript. RCM contributed to study design and provided critical revisions. CCH was involved in study design, acquisition and analysis of data, and critical review of the manuscript. SB helped with acquisition and analysis of data and critical review of the manuscript. TMP contributed to interpretation of data and provided critical revisions. DL was involved with interpretation of data and critical review of the manuscript. RHH provided analysis and interpretation of data and critical revisions. JZ contributed to acquisition and analysis of data and critical review of the manuscript. JMW was involved in analysis and interpretation of data and critical review of the manuscript. MGH helped with study design and interpretation of data and provided critical revisions. JHD participated in interpretation of data and critical review of the manuscript. RDS provided interpretation of data and critical revisions. CLW contributed to study design, interpretation of data, and critical review of the manuscript. JLC participated in study design, interpretation of data, and critical revisions. JMH contributed to study conception and design, interpretation of data, and drafting of the manuscript. All authors have read and approved the final manuscript.
